# Unilateral acute posterior multifocal placoid pigment epitheliopathy in a convalescent COVID-19 patient

**DOI:** 10.1186/s40942-021-00312-w

**Published:** 2021-05-25

**Authors:** Francisco Olguín-Manríquez, Linda Cernichiaro-Espinosa, Arturo Olguín-Manríquez, Rebeca Manríquez-Arias, Erick Omar Flores-Villalobos, Perla Ayumi Kawakami-Campos

**Affiliations:** 1 Retina Department, Oftal Unidad Médica, Mexico city, Mexico; 2 Retina Department, Asociación Para Evitar La Ceguera en México (APEC), Mexico City, Mexico; 3 Cornea and Anterior Segment Department, Oftal Unidad Médica, Pachuca de Soto, Hidalgo Mexico; 4 General Ophthalmologist, Oftal Unidad Médica, Pachuca de Soto, Hidalgo Mexico; 5 Retina Department, Centro Oftalmológico de Alta Especialidad (COFAE), Pachuca de Soto, Hidalgo Mexico; 6 Retina Department, Instituto Nacional de Ciencias Médicas Y Nutrición “Salvador Zubirán”, Mexico City, Mexico; 7 Retina Service, Oftal Unidad Médica, Av. Paseo de la Reforma 155, Colonia Lomas de Chapultepec, Alcaldía Miguel Hidalgo, 11000 Mexico City, CP Mexico

**Keywords:** Acute posterior multifocal placoid pigment epitheliopathy, Severe acute respiratory syndrome coronavirus 2, Coronavirus disease 2019

## Abstract

**Background:**

To report a case of unilateral acute posterior multifocal placoid pigment epitheliopathy (APMPPE) in a Hispanic convalescent COVID-19 female patient.

Case presentation

A 35-year-old Hispanic female with exposure to the severe acute respiratory syndrome coronavirus 2 (SARS-CoV-2) was evaluated due to unilateral visual loss. Ophthalmic examination and diagnostic tests were consistent with APMPPE.

**Discussion:**

Ocular changes can be observed in patients with COVID-19. A complete ophthalmic evaluation must be performed in patients with low vision after SARS-CoV-2 infection.

## Introduction

In February 2020, the International Committee on Taxonomy of Viruses (ICTV) announced the severe acute respiratory syndrome coronavirus 2 (SARS-CoV-2) as a new virus. This name was chosen because the virus is genetically related to the coronavirus responsible for the SARS outbreak of 2003. The World Health Organization (WHO) named this new disease as "COVID-19" in February 2020. The disease quickly reached the size of a pandemic on March 11, 2020, due to the speed and scale of the transmission [1].

There are basically 3 stages or phases in the natural history of COVID-19, regarding disease severity. The first phase is related to the onset of the disease and is generally characterized by the development of influenza-like symptoms from mild to moderate. Some individuals recover and some progress to the second phase. During the second phase, it is possible to detect pneumonia-like symptoms. Depending on the severity of phase two, patients can improve, or worsen with the necessity of intubation and ventilation. The phase three is characterized by hyper inflammation and sepsis of lungs. Patients often require intensive care unit and most of them unfortunately cannot overcome the infection and eventually die [2].

Ocular manifestations in COVID-19 patients may include conjunctivitis, chemosis, and epiphora. Higher neutrophil counts, procalcitonin, C-reactive proteins, and lactate dehydrogenase are factors associated with such signs [3].

Posterior segment manifestations may include retinal hemorrhages, cotton wool spots, and dilated retinal vessels. Higher veins diameter has been positively correlated with disease severity [4]. Interestingly, SARS-CoV-2 infects endothelial cells [5]. Hyper-reflective lesions at the level of inner plexiform and ganglion cell layers have been documented in twelve patients examined 11–33 days after the onset of COVID-19 symptoms [6]. Vitritis and disruption of the ellipsoid zone and hyper-reflective lesions at the level of inner plexiform and ganglion cell layers have also been reported [7].

We present a case of a convalescent COVID-19 female patient with acute posterior multifocal placoid pigment epitheliopathy (APMPPE).

## Case presentation

A 35-year-old Hispanic female presented blurred vision, central scotoma and photopsia in her right eye. She had experienced fever, headaches and myalgia 6 weeks before initial consultation. The patient had past history of Diabetes mellitus diagnosed 3 years before, and good glucose control was obtained with insulin. She tested positive for SARS-Cov-2 IgG antibody 2 weeks before ophthalmic examination. Previous ocular history was unremarkable.

**Figure. 1:**
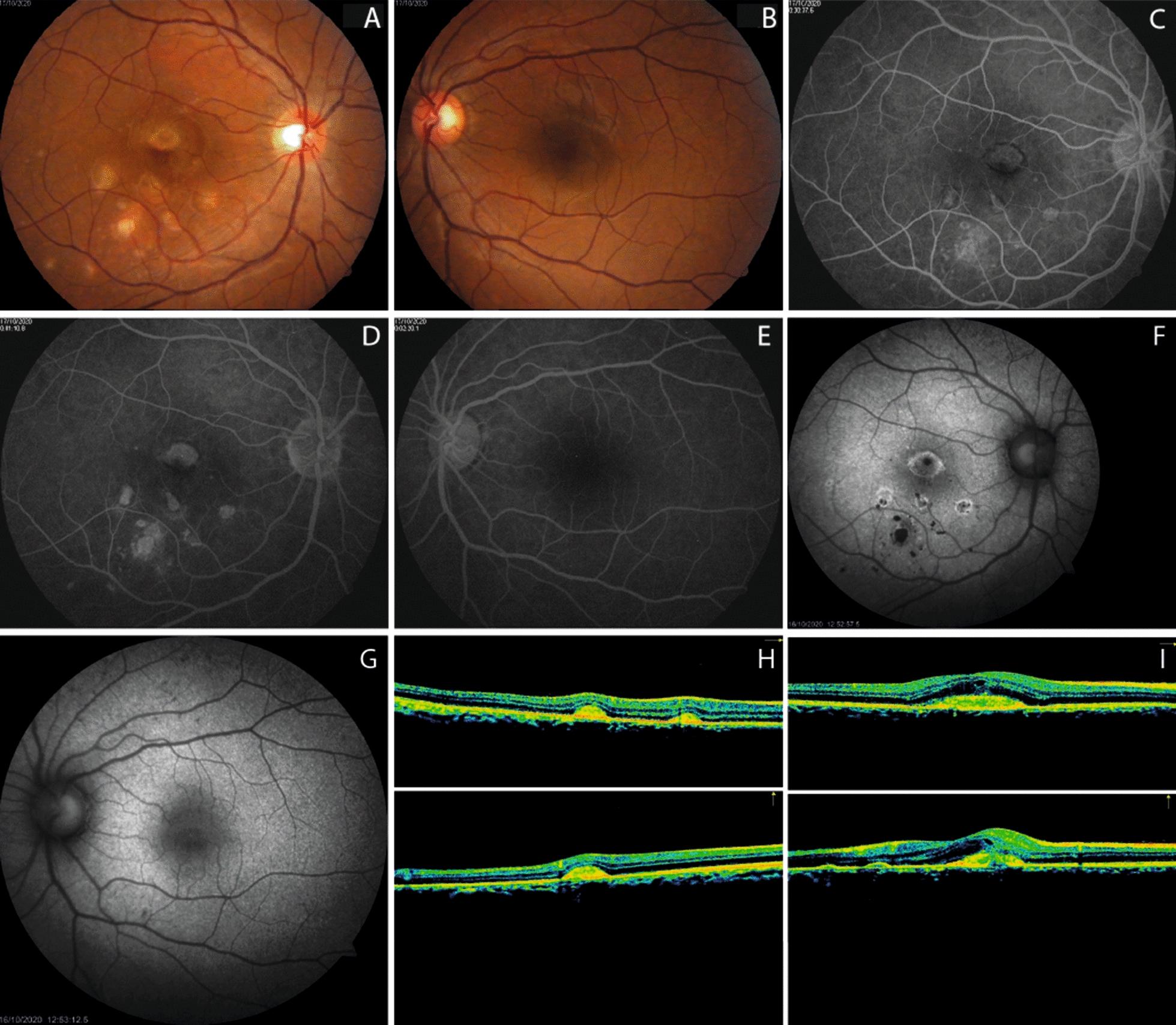
Fundus multimodal imaging of a patient with serological evidence of SARS-CoV-2 exposure. **A**, **B**: color fundus photographs showing multiple yellow–white placoid lesions involving the fovea in the right eye. Left eye posterior segment is unremarkable. **C–D**: fluorescein angiography demonstrates early hypofluorescence with hyperfluorescent staining of all lesions. **E**: normal fluorescein angiogram in the LE. **F**: fundus autofluorescence showing hypoautofluorescent lesions surrounded by a hyperautofluorescent halo in the right eye. **G**: fundus autofluorescence shows isoautofluorescence in the left eye. **H–I**: cross-sectional optical coherence tomography (OCT) scans in extrafoveal lesions demonstrate hyper-reflective material at the level of the outer retinal layers. Disruption of the interdigitation zone and hyper-reflective material is observed at the foveal depression

A complete ophthalmological exam was performed. Best-corrected visual acuity (BCVA) was 20/400 in the right eye (RE) and 20/20 in the left eye (LE). Biomicroscopy of the anterior segment, pupillary reflexes, and intraocular pressure were normal in both eyes (OU). At fundus examination, multiple yellow-white placoid lesions were evident at the posterior pole in the RE (Fig. 1a). LE posterior segment was unremarkable (Fig. 1b). Fluorescein Angiography (FA) showed early hypofluorescence at the foveal lesions with late hyperfluorescence of all lesions (Fig. 1c–d). Fundus autofluorescence (FAF) revealed hypo-autofluorescence of all lesions surrounded by a hyper-autofluorescent halo (Fig. 1f). Optical Coherence Tomography (OCT) scans across the placoid lesions showed hyper-reflective material at the level of the outer retinal layers and disruption at the interdigitation zone in the foveal depression (Fig. 1h–i).

Complete blood count with differential and tests for infectious disease (tuberculosis, syphilis, and toxoplasmosis) were requested—the results obtained were negative. Routine blood tests were normal.

Based on imaging and clinical features, the patient was diagnosed with APMPPE.

## Discussion

More commonly ocular manifestations have been reported in patients with COVID-19 such as conjunctivitis, chemosis, and epiphora in patients with severe systemic disease or abnormal findings on blood tests [3].

Our patient started with unilateral macular dysfunction 6 weeks after the onset of influenza-like symptoms. Laboratory tests found positive IgG antibody for SARS-CoV-2, showing serological evidence of past infection for COVID-19. Fundus photographs, FA, FAF, and OCT findings correlate well with previous descriptions of APMPPE lesions [14, 15]. APMPPE is an acute-onset inflammatory disease that affects the choriocapillaris, RPE, and outer retina. Numerous, yellow, creamy colored placoid lesions are seen in the posterior pole and are not seen anterior to the equator [13].

Up to one third of APMPPE cases worldwide have been described as related to a viral prodromic period. While viruses as type five Adenovirus and B4 Coxsackievirus have been typically associated with white dot syndromes, there have been reports of post vaccination disease. Its cause has not been clearly elucidated yet since the incidence of these syndromes is very low. However, Coxsackie antibody titers in acute and treated cases seem to correlate in some series [16].

Using multimodal imaging in APMPPE, some authors have found choroidal inflammation as a preceding and necessary factor for RPE, and subsequent damage in cones [16]. This choroidal inflammation may be generated solely by the entry of SARS-CoV-2 via the angiotensin-converting enzyme 2 receptor (ACE) in the endothelium. The presence and abundance of this receptor has already been found higher in COVID-19 fatal cases compared to controls [17]. In these patients the accumulation of mononuclear cells and neutrophils in the lumen and intima of choriocapillaris may prove the viral trigger for white dot syndromes.

Detection of SARS-CoV-2 has been confirmed by Real-time reverse transcriptase-polymerase chain reaction (RT-PCR) in human retinal biopsies (RB) of deceased COVID-19 patients. Although ocular symptoms or changes in the posterior segment were not analyzed in these patients, important information about virus presence in the retina was confirmed [8].

Experimental coronavirus retinopathy (ECOR) occurs after intravitreal inoculation of murine coronavirus (M-CoV, JHM strain) in BALB/c and CD-1 mice. In BALB/c mice, JHM strain induces a biphasic retinal disease characterized by retinal vasculitis observed one to seven days after inoculation (early phase) followed by retinal degeneration in the absence of inflammation (second phase) [9]. The virus can be observed intracellularly within vacuoles and extracellularly at the plasma membrane. [10] The reduction in the interphotoreceptor retinoid-binding protein (IRBP) is a proposed mechanism of retinal damage observed in ECOR models. IRBP is synthesized by retinal photoreceptor cells and acts as an important transport of retinoids between photoreceptors and RPE. The coronavirus could interfere with normal synthesis and/or secretion of IRBP through the rough endoplasmic reticulum and Golgi complex of the photoreceptor cell. Thus, the resultant retinoid excess in the photoreceptor can be toxic [11]. Further investigations found a correlation between higher levels of Interferon-_ϒ_ (IFN-_ϒ_) and retinal damage in BALB/c mice after inoculation of JHM strain in the vitreous cavity [12].

There is uncertainty if retinal changes (micro hemorrhages, cotton wool spots) or changes on OCT (hyper reflective dots at the level of inner plexiform or ganglion cell layers) appear days or weeks after the onset of COVID-19 and its relation to the severity of the disease or symptoms [4, 6, 7].

To the best of our knowledge, this is the first case of APMPPE in a convalescent COVID-19 patient. Understanding of the physiopathology is still needed15.

## Data Availability

The data sets used and analyzed during the current study are available from the corresponding author on reasonable request.

## Abbreviations

APMPPE: Acute posterior multifocal placoid pigment epitheliopathy
WDS: White dot syndromes
COVID-19: Coronavirus disease
WHO: World health organization
RPE: Retinal pigment epithelium
OCT: Optical coherence tomography
FAF: Fundus autofluorescence
FA: Fluorescein angiography
RE: Right eye
LE: Left eye
OU: Both eyes
IRBP: Interphotoreceptor retinoid-binding protein
